# Pretreatment creatinine-to-cystatin c ratio as a prognostic marker in digestive tract cancers: a meta-analysis with reconstructed individual patient data and trial sequential analysis

**DOI:** 10.3389/fnut.2026.1834663

**Published:** 2026-07-24

**Authors:** Yongping Hao, Wenhui Li, Guangwei Tian, Nan Li

**Affiliations:** 1 Department of Radiation Oncology, The First Affiliated Hospital of China Medical University, Shenyang, China; 2 Laboratory of Gastrointestinal Onco-Pathology, Cancer Institute and General Surgery Institute, The First Affiliated Hospital of China Medical University, Shenyang, China

**Keywords:** creatinine-to-cystatin C ratio, digestive tract cancers, prognosis, reconstructed individual patient data, sarcopenia

## Abstract

**Background:**

Sarcopenia, characterized by loss of skeletal muscle, is associated with unfavorable clinical outcomes in patients with cancer. The creatinine-to-cystatin C ratio (CCR) has emerged as a readily available marker related to muscle reserve. This meta-analysis aimed to examine the prognostic utility of baseline CCR levels in patients with digestive tract cancers.

**Methods:**

We searched PubMed, Embase, Web of Science, and the Cochrane Library from database inception through 22 October 2025. Original cohort studies that enrolled adults with histologically proven digestive tract cancers, reported pretreatment CCR with overall survival hazard ratios, and classified patients using a specific CCR cut-off were included. Risk of bias was evaluated with the Newcastle-Ottawa Scale and the Quality in Prognostic Studies tool. Pooled hazard ratios for overall survival were synthesized. Patient-level survival data were reconstructed from published Kaplan-Meier curves. No original patient-level datasets were obtained from study investigators. Trial sequential analysis (TSA) was performed as an exploratory robustness assessment.

**Results:**

Nine independent cohorts comprising 3,073 patients were included. Lower CCR was significantly associated with shorter OS (HR = 1.71; 95% CI: 1.45–2.02; *p* < 0.001). Stratified analyses showed consistent prognostic associations in esophageal cancer (HR = 2.21; 95% CI: 1.35–3.61; *p* = 0.002), biliary tract cancer (HR = 2.62; 95% CI: 1.31–5.23; *p* = 0.006), and gastrointestinal cancers (HR = 1.65; 95% CI: 1.34–2.05; *p* < 0.001). Reconstructed IPD analysis also showed shorter OS in the low-CCR group (HR = 1.48; 95% CI: 1.32–1.67; *p* < 0.001). Exploratory trial sequential analysis was consistent with the pooled OS association under prespecified assumptions.

**Conclusion:**

Pretreatment CCR was associated with overall survival in patients with digestive tract cancers. However, further prospective studies are needed to confirm these findings.

**Systematic review registration:**

https://www.crd.york.ac.uk/prospero/, identifier CRD420261290556.

## 1 Introduction

Sarcopenia, characterized by a sustained loss of skeletal muscle mass, not only predicts reduced treatment tolerance but is also closely linked to increased infection rates, prolonged hospital stays, and shorter survival in patients with cancer (1–3). The third-lumbar skeletal muscle index (SMI), measured by computed tomography (CT) or dual-energy X-ray absorptiometry (DXA), is regarded as the gold standard for quantifying muscle mass but is costly and requires specialized equipment and trained technicians, limiting its accessibility in large-scale oncologic populations (4).

Recently, the creatinine-to-cystatin C ratio (CCR) has been proposed as a surrogate marker related to muscle mass. This is based on the biological distinction that creatinine is produced exclusively by skeletal muscle, whereas cystatin C is synthesized by all nucleated cells at a constant rate (5, 6). Since the CCR can be calculated from routine blood tests, it offers a simple, minimally invasive, inexpensive, and repeatable marker for assessing muscle reserve in cancer patients (7, 8).

Several studies have already linked a pre-treatment decrease of CCR to unfavorable outcomes in gastrointestinal and other malignancies. In a large retrospective cohort of 3,060 solid-tumor patients, Jung et al. demonstrated that a lower CCR was strongly associated with higher short-term mortality (9). Comparable findings have been reported for esophageal cancers and gastrointestinal stromal tumors. Even after adjustment for conventional TNM stage, performance status, and systemic inflammatory markers, CCR retained independent prognostic value (10, 11). Nevertheless, existing studies are highly heterogeneous in sample size and cut-off definition, and a quantitative synthesis of its prognostic value for overall survival remains unavailable. We therefore performed a meta-analysis to synthesize the available evidence and clarify the prognostic significance of the CCR in patients with digestive tract cancers, providing an evidence-based foundation for future screening and intervention strategies.

## 2 Materials and methods

The methodological framework and reporting procedures of this study were formulated in accordance with the Preferred Reporting Items for Systematic Reviews and Meta-Analyses 2020 (PRISMA 2020) statement (12) and the Assessing the Methodological Quality of Systematic Reviews (AMSTAR) guideline (13). The study protocol was registered with PROSPERO (registration number: CRD420261290556).

### 2.1 Study design and samples

To evaluate the prognostic value of CCR in digestive tract cancers, we designed a systematic review and meta-analysis that included trial sequential analysis (TSA). Literature retrieval was performed across PubMed, Cochrane Library, EMBASE, and Web of Science from database inception through 22 October 2025, with no language restrictions. The core search combined CCR terms (“creatinine/cystatin C ratio” or “creatinine to cystatin C ratio” or “creatinine over cystatin C ratio” or “creatinine-to-cystatin C ratio” or “sarcopenia index” or “creatinine-cystatin c ratio”) with cancer terms (“carcinoma” or “cancer” or “tumor” or “tumour” or “neoplasm”), and the strategy was adapted for each database (Supplementary Table 1). We included original articles that (1) enrolled adults with histologically proven digestive-tract neoplasms, (2) provided pretreatment CCR together with a hazard ratio (HR) and 95% confidence interval (CI) for survival outcome and (3) split participants into two groups based on a specific CCR cut-off. Editorials, reviews, conference abstracts, posters, and studies lacking survival information were excluded. This analysis was performed based on previously published studies. Thus, ethical approval was not required.

### 2.2 Study variables

The main exposure was the CCR, and the primary outcome endpoint was OS, analyzed using pooled HRs with 95% CIs. Heterogeneity across cohorts was quantified with the I^2^ statistic. Exploratory subgroup analyses were based on publication year, cancer type, study design, country, sample size, CCR cut-off value, and the method of cut-off selection. We also recorded sex distribution, renal exclusion criteria, renal-function adjustment where reported, and covariates included in the original adjusted models.

### 2.3 Data collection and analysis

Records identified from PubMed, Cochrane Library, EMBASE, and Web of Science were imported into EndNote (Clarivate, Philadelphia, PA, United States), and duplicates were removed. Two authors independently screened the literature and extracted data. Any discrepancies were settled by discussion. The following items were recorded: first author, publication year, tumor type, country, study design, sample size, CCR cut-off and its selection method, survival endpoint, age, sex distribution, treatment, follow-up duration, stage, renal-function exclusion or adjustment where reported, and the hazard ratio with 95% CI for survival outcome. Cohort quality was appraised using the Newcastle-Ottawa Scale (NOS) across three domains: selection, comparability, and outcome. In addition, risk of bias in included prognostic cohorts was assessed using the Quality In Prognostic Studies (QUIPS) tool (14). Two authors independently assessed each cohort.

The statistical analysis was carried out with Stata 12.0 (StataCorp, College Station, TX) and R 3.6.1. HRs together with 95% CIs were pooled, and heterogeneity was evaluated with Cochran’s Q and the I^2^ statistic (15). To ensure consistency in the direction of effect estimates, all HRs were standardized to represent the comparison of the low-CCR group versus the high-CCR group (low vs. high). For studies that originally reported HRs in the reverse direction (high vs. low), the HR and its 95% CI were inverted by taking their reciprocals.

Heterogeneity was considered significant for *p*-values < 0.1 or *I*^2^ > 50%. Under these conditions, a random-effects model was used. Subgroup analyses and meta-regression were exploratory because of the limited number of cohorts. Sensitivity analysis was conducted to evaluate the stability of the result. Publication bias was assessed using Begg’s test (16), Egger’s test (17), and the trim-and-fill method (18). Because fewer than 10 cohorts were included, these tests were interpreted cautiously as exploratory assessments. Trial sequential analysis (TSA) was performed as a complementary exploratory robustness assessment in R 3.6.1, invoked from Stata 12.0. A 15% relative risk reduction (RRR) was prespecified as the anticipated effect size for calculating the required information size, consistent with the conventional TSA (15%) scenario (19) and within the pragmatic 10–20% range suggested when prior evidence is limited (20). TSA was carried out using alpha = 5%, beta = 20% (80% power). Since TSA was originally developed for intervention meta-analyses and all included cohorts were observational prognostic studies, the TSA result was interpreted as exploratory rather than definitive evidence that no further validation is needed.

The digitalization of published Kaplan-Meier (KM) graphs was accomplished using Engauge Digitizer 11.3. Reconstruction of individual patient data (reconstructed IPD) was then performed using the IPDfromKM method (21). The extracted data included the KM curves, the corresponding number of at-risk patients at each time point, and overall survival outcomes for the entire study population in each included study. Each reconstructed KM curve was reviewed to verify its consistency with the original graphical representation. Survival analyses were performed in R Studio 2025.09.2 using the survival, survminer, and ggsurvfit packages. Since the reconstructed IPD did not contain patient-level stage, performance status, renal function, inflammatory markers, nutritional status, or treatment covariates, Cox models based on reconstructed IPD were unadjusted. Statistical significance was prespecified at *P* < 0.05.

## 3 Results

### 3.1 Literature search and characteristics of the included cohorts

**FIGURE 1:**
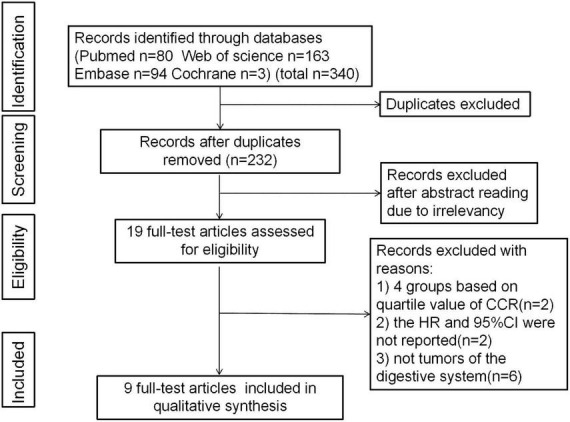
Flow diagram of the study selection procedure.

Systematic screening identified 340 database records (PubMed, *n* = 80; Web of Science, *n* = 163; Embase, *n* = 94; Cochrane Library, *n* = 3). After removal of 108 duplicates, 232 records were screened by title and abstract, 19 full-text articles were assessed for eligibility, and 9 independent cohorts including 3,073 patients were included (Supplementary Table 2). The selection flow chart is shown in Figure 1. Eight studies were conducted in Chinese populations and one in Japan. All cohorts were retrospective. The 9 cohorts covered four digestive tract cancer categories: gastrointestinal tumors (four cohorts) (22–25), esophageal carcinoma (two cohorts) (11, 26), cholangiocarcinoma or biliary tract cancer (two cohorts) (27, 28), and pancreatic cancer (one cohort) (29). Study quality was assessed with the Newcastle-Ottawa Scale. Study characteristics, including sex distribution, renal-function handling, and adjusted overall survival estimates reported by the original cohorts, are summarized in Table 1. Detailed covariate extraction and verification notes are provided in Supplementary Table 3. Domain-level NOS scores are provided in Supplementary Table 4, and QUIPS domain-level risk-of-bias judgments are provided in Supplementary Table 5.

**TABLE 1:** Characteristics of the included studies.

References	Study	Year	Cancer type	Country	Design	N	CCR cut-off	Cut-off method		Outcome
Feng et al. (11)	Feng	2025	Esophageal cancer	China	Retrospective	202	97.5 (Unit unknown)	Restricted cubic spline		OS
Gao et al. (22)	Gao	2023	Colorectal cancer	China	Retrospective	975	1.0675	The optimal layering method		OS/PFS
Sun et al. (23)	Sun	2022	Gastric cancer	China	Retrospective	327	0.67	ROC curve		OS
Ji et al. (24)	Ji	2024	Metastatic gastric cancer	China	Retrospective	113	0.7148	median value		OS/PFS
Liu et al. (25)	Liu	2023	Gastrointestinal cancer	China	Retrospective	699(OS), 688(DFS)	Unknown	ROC curve		OS/DFS
Zheng et al. (26)	Zheng	2022	Esophageal cancer	China	Retrospective	203	0.62(men) 0.55(women)	ROC curve		OS
Fukushima et al. (27)	Fukushim a	2024	Biliary tract cancer	Japan	Retrospective	190	0.848	ROC curve		OS/RFS
Liu et al. (28)	Liu	2024	Biliary tract cancer	China	Retrospective	149	1.0834	ROC curve		OS/RFS
Bi et al. (29)	Bi	2024	Pancreatic cancer	China	Retrospective	215	1.09	ROC curve		OS/DFS
**References**	**Age**	**Treatment**	**Follow-up (months)**	**Stage**	**Analysis**	**Sex (M/F)**	**Renal function handling**	**Adjusted OS HR (95% CI)**	**NOS**	**QUIPS overall**
Feng et al. (11)	64(mean) (45–75)	Surgery	40(7–54)	Mixed	Multivariable Cox	182/20	Not reported	0.482 (0.271–0.857)	8	Moderate
Gao et al. (22)	57.50(mean) ±13.14	Surgery	72.8(38.9–88.4)	III-IV	Multivariable Cox	607/368	Severe renal insufficiency excluded	1.410(1.087–1.829)	9	Moderate
Sun et al. (23)	64(median) (20–89)	Surgery	Unknown	Mixed	Multivariable Cox	234/93	eGFR <60 mL/min/1.73 m^2^ excluded (*n*=12)	0.565 (0.311–1.025); *p* = 0.060	8	Moderate
Ji et al. (24)	63(median) (57–69)	Non-surgery	Unknown	IV	Multivariable Cox	96/17	eGFR <60 mL/min/1.73 m^2^ excluded	2.528 (1.317–4.854)	7	Moderate
Liu et al. (25)	63.07(mean) ±10.89	Unknown	Unknown	Unknown	KM	630/325	Acute kidney injury excluded	KM plot HR 0.34 (0.17–0.68)	6	High
Zheng et al. (26)	66.7(mean)	Surgery	7(0–23)	Mixed	Multivariable Cox	162/41	No explicit renal function exclusion in validation cohort	2.62(1.02–6.77)	8	Moderate
Fukushima et al. (27)	73(median) (39–89)	Surgery	Unknown	Unknown	Multivariable Cox	NR	Not reported	2.60 (1.07–6.29)	8	Moderate
Liu et al. (28)	64.3(mean) ± 7.8	Surgery	Unknown	Unknown	Multivariable Cox	85/64	Not reported	2.654(0.880–8.004); *p*=0.083	7	Moderate
Bi et al. (29)	61.3(mean) ± 9.1	Surgery	Unknown	Mixed	Multivariable Cox	NR	Not reported	1.459(1.026–2.076); *p*=0.036	8	Moderate

OS, overall survival; DFS, disease-free survival; RFS, recurrence-free survival; PFS, progression-free survival; NR, not reported in accessible source; NOS, Newcastle-Ottawa Scale; RCS, restricted cubic spline; ROC, receiver operating characteristic; KM, Kaplan-Meier; Cox, Cox proportional hazards model.

### 3.2 Prognostic value of CCR in digestive tract cancers

**FIGURE 2:**
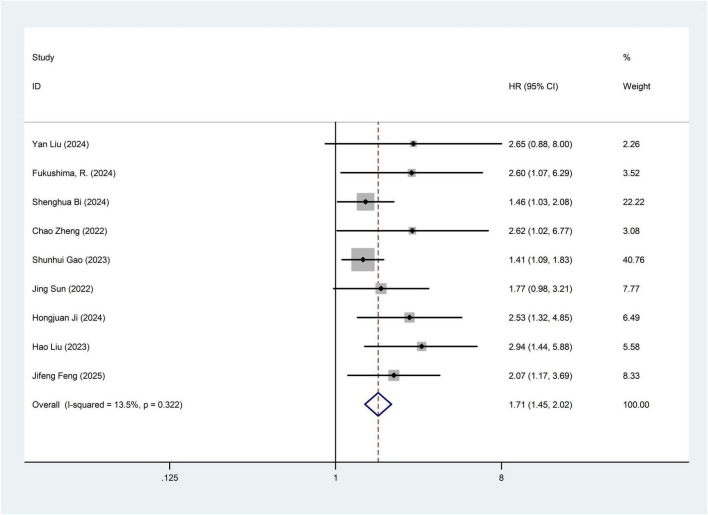
Forest plot of the HR for the association between CCR and OS in digestive tract cancers. HR, hazard ratio; CCR, creatinine-to-cystatin C ratio; OS, overall survival.

OS data were available for 3,073 patients from 9 cohorts. Patients with a lower CCR had significantly shorter OS (HR = 1.71; 95% CI: 1.45–2.02; *p* < 0.001) than those with a higher CCR (Figure 2). No significant statistical heterogeneity was detected (*I*^2^ = 13.5%; *p* = 0.322), and a fixed-effect model was used. However, this statistical homogeneity does not eliminate clinically relevant differences among cancer types.

**FIGURE 3:**
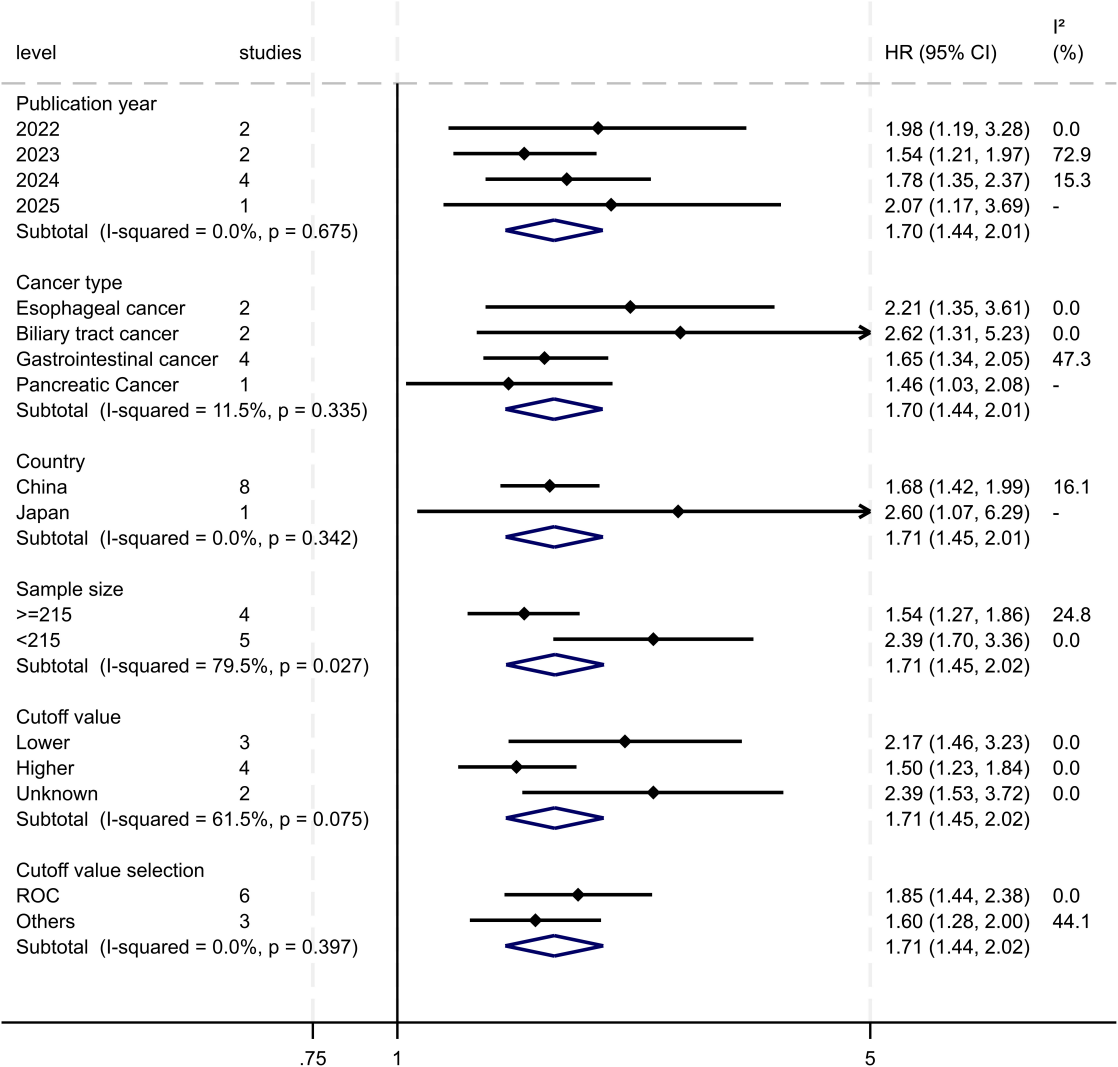
Forest plot of subgroup analyses for the association between CCR level and overall survival (OS).

Subgroup analyses of the 9 cohorts contributing to OS were performed by publication year, tumor type, study design, country, sample size, CCR cut-off value, and cut-off selection method (Figure 3). Tumor-specific stratification showed that higher CCR remained associated with better OS in gastrointestinal tumors (HR = 1.65; 95% CI: 1.34–2.05; *p* < 0.001; four cohorts), biliary tract cancer (HR = 2.62; 95% CI: 1.31–5.23; *p* = 0.006; two cohorts), and esophageal cancer (HR = 2.21; 95% CI: 1.35–3.61; *p* = 0.002; two cohorts), with no significant statistical heterogeneity across cancer types (Supplementary Figure 1). Pancreatic cancer was represented by a single cohort (Bi 2024; Ref 29; *n* = 215), whose adjusted HR was 1.459 (1.026–2.076; *p* = 0.036). A tumor-type pooled estimate was therefore not calculated for this category. Reported cut-offs ranged from 0.55 to 1.09, indicating that no standardized clinical threshold can yet be recommended. These subgroup analyses were interpreted as exploratory because the number of cohorts within each subgroup was small.

Given the limited number of cohorts (*n* = 9), we performed exploratory meta-regression for publication year, sample size, CCR cut-off type and cancer type. The results showed no statistically significant moderator effects (all *P* > 0.05) (Supplementary Table 6), consistent with the low between-study heterogeneity (*I*^2^ = 13.5%).

### 3.3 Sensitivity analysis and publication bias

**FIGURE 4:**
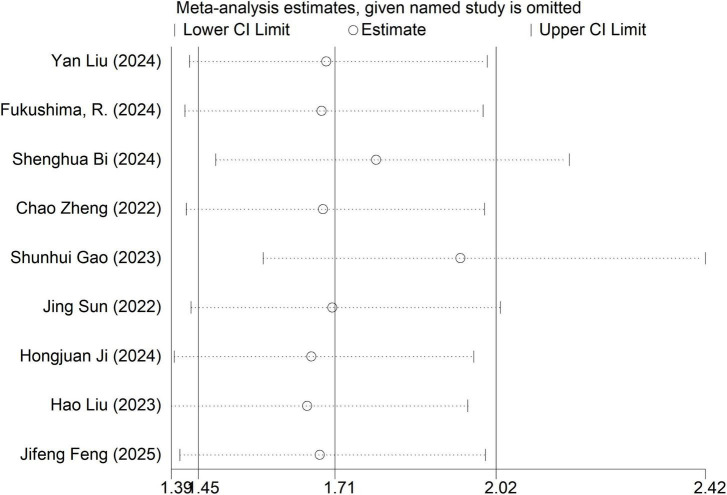
Sensitivity analysis of the association between CCR and OS in digestive tract cancers.

Sensitivity analyses were performed to evaluate the stability of the pooled estimates. Removal of any single study did not materially alter the overall HR (Figure 4).

**FIGURE 5:**
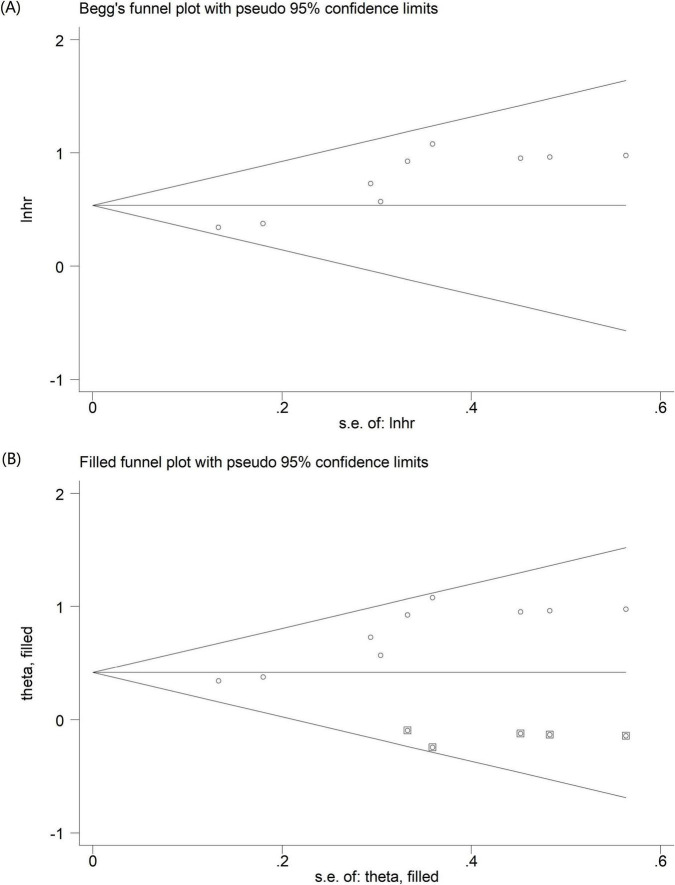
Test results for publication bias. **(A)** Begg’s test. **(B)** Trim-and-fill method.

To examine potential publication bias, we used Begg’s funnel plot and Egger’s linear regression test. An asymmetric funnel distribution was observed (Figure 5A), and Egger’s test was significant (*p* < 0.001), suggesting possible small-study effects or publication bias regarding OS. Following the trim-and-fill methodology to impute 5 potentially missing studies, the association between CCR and OS remained statistically significant (*p* < 0.001) (Figure 5B). This analysis should be considered exploratory rather than definitive evidence of robustness.

### 3.4 Trial sequential analysis

**FIGURE 6:**
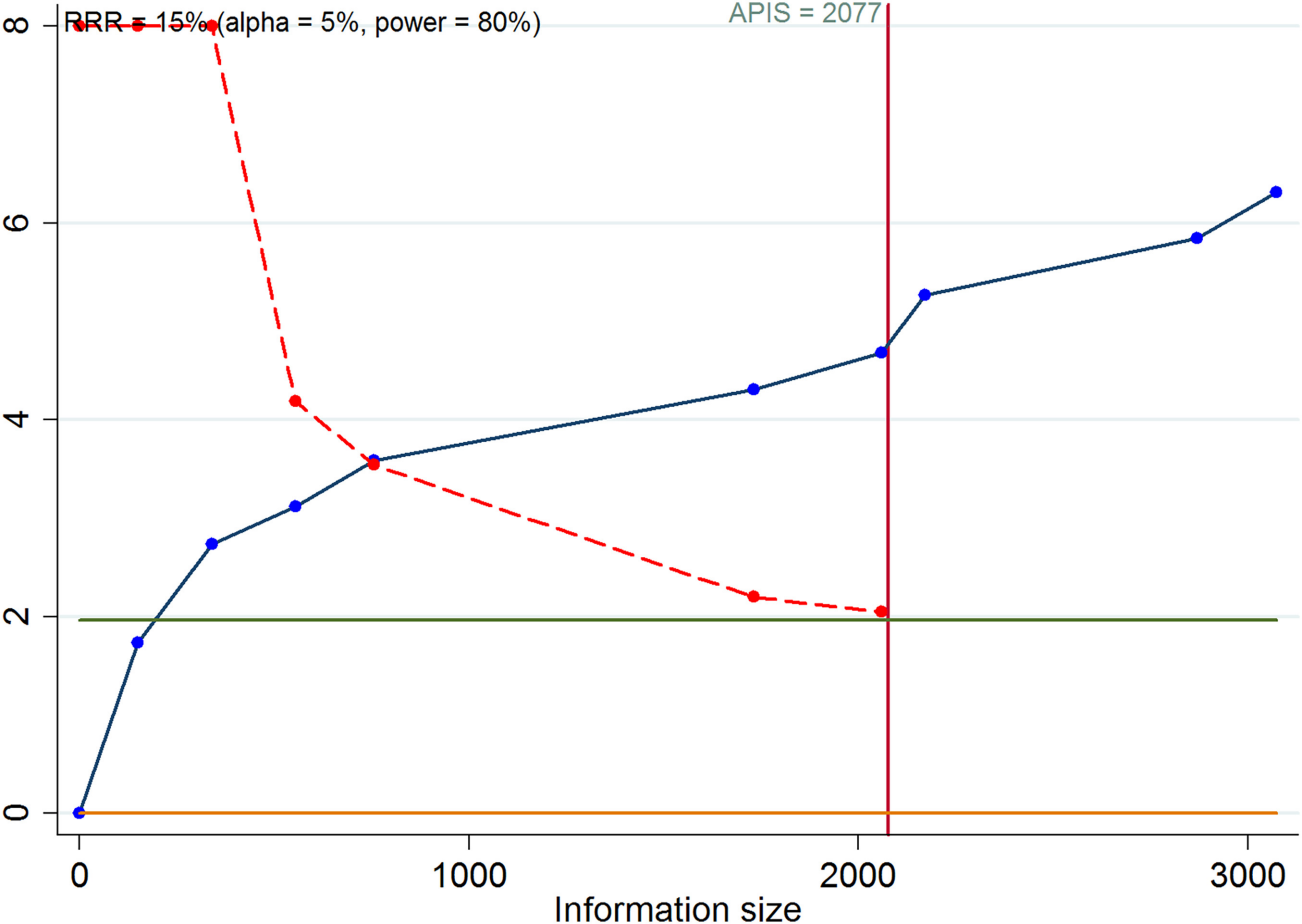
Trial sequential analysis (TSA) between CCR and OS. Alpha = 5%; beta = 20%; relative risk reduction (RRR) = 15%. CCR, creatinine-to-cystatin C ratio; OS, overall survival.

With parameters set at 15% relative risk reduction, type I error probability of 5%, and 80% power, TSA was performed to assess whether the accrued evidence crossed sequential monitoring boundaries. The cumulative Z curve crossed the monitoring boundary for CCR predicting OS in digestive tract cancers (Figure 6). This result supports the stability of the observed association under the prespecified assumptions. Further prospective validation is still needed.

### 3.5 Reconstructed IPD survival analysis

**FIGURE 7:**
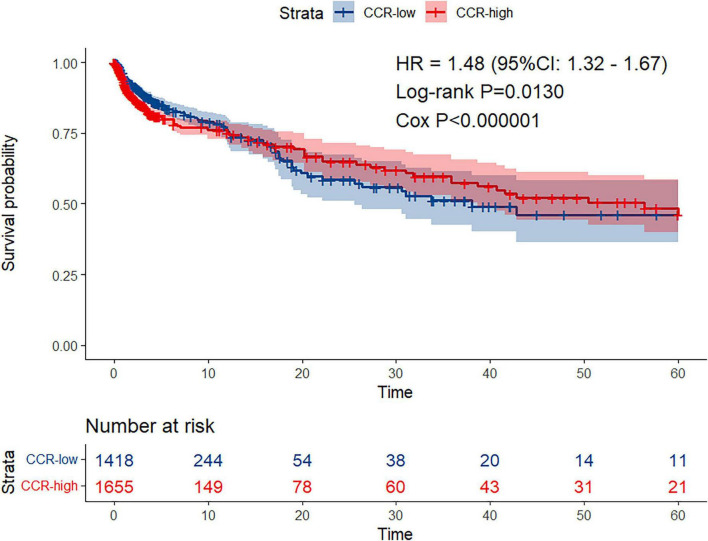
KM curves for OS reconstructed from published Kaplan-Meier curves, stratified by CCR status.

Reconstructed individual patient data (reconstructed IPD) were obtained for 3,073 patients with evaluable baseline CCR status across the 9 included cohorts. The study population comprised 1,655 patients (53.9%) in the high-CCR group and 1,418 (46.1%) in the low-CCR group. Kaplan-Meier curves generated from the reconstructed IPD showed a sustained and early separation of survival distributions favoring the CCR-high group (Figure 7). Specifically, patients with low CCR had significantly shorter OS than those with high CCR (HR = 1.48; 95% CI: 1.32–1.67; *p* < 0.001) (Figure 7). Because patient-level covariates were unavailable, this reconstructed-IPD Cox analysis should be interpreted as an unadjusted survival comparison. Notably, this patient-level HR was smaller than the pooled study-level estimate (HR = 1.71), possibly because the meta-analysis combined cohort-specific hazard ratios, mostly from multivariable models in the original studies, whereas the reconstructed-IPD analysis used a single unadjusted Cox model. Both analyses showed the same direction of association with overlapping confidence intervals.

## 4 Discussion

This meta-analysis synthesized data from nine independent cohorts to evaluate the prognostic significance of CCR in digestive tract malignancies. Elevated CCR levels were associated with prolonged OS across the pooled datasets. Subgroup analyses suggested a consistent direction of association across gastrointestinal, biliary tract, esophageal, and pancreatic cancers, although the small number of cohorts within each subgroup limits tumor-specific inference. To our knowledge, this is the first meta-analysis to quantify the association between CCR and long-term survival outcomes in patients with digestive tract cancer using both aggregate HRs and reconstructed time-to-event data.

In prior single-cohort studies, CCR has served as a clinically accessible surrogate for skeletal muscle mass and an independent prognostic indicator in digestive tract malignancies. Given that gastrointestinal cancers frequently induce severe cachexia and muscle wasting, objective assessment of muscle reserves is critical for predicting treatment tolerance and survival outcomes. Recent studies have demonstrated that CCR closely correlates with CT-quantified skeletal muscle index (SMI) and psoas muscle area, establishing its validity as a non-invasive measure of muscle reserves in cancer populations (24, 26, 30). Pretreatment elevated CCR was independently associated with reduced incidence of severe postoperative complications, shorter length of hospital stay, and lower readmission rates following major cancer treatments, reflecting preserved physiological reserve and superior functional status (31, 32). However, CCR should not be interpreted as a standalone diagnostic test for sarcopenia. Serum creatinine reflects both muscle mass and glomerular filtration, so renal dysfunction can alter CCR independently of muscle reserve (6). Cystatin C is less muscle-dependent but may increase with systemic inflammation. Meta-analytic and mechanistic data suggest cytokine-mediated up-regulation, including IL-6-related pathways, and other non-renal conditions can raise cystatin C independent of glomerular filtration (7). In patients with cancer, malnutrition and cachexia-related muscle loss may lower creatinine generation, whereas inflammatory activation and treatment exposure may further disrupt the concentrations of both analytes (7, 9). These non-muscular determinants may therefore weaken the specificity of CCR as a muscle-reserve index.

Several recent studies have specifically examined the prognostic utility of CCR in gastrointestinal malignancies. In metastatic gastric cancer, Ji et al. demonstrated that elevated pretreatment CCR independently predicts a favorable response to PD-1 inhibitor-based combination therapy, with higher baseline levels associated with improved objective response rates and prolonged survival (24). Similarly, in colorectal cancer, Gao et al. established CCR as an independent prognostic indicator, with higher levels correlating with reduced recurrence risk, lower mortality, and shorter hospitalization duration (22). These results collectively endorse CCR as a readily available clinical biomarker for prognostic stratification and survival forecasting in digestive tract malignancies. However, further prospective multicenter studies are needed to establish standardized cut-offs and to confirm these findings.

Several limitations warrant consideration. First, the optimal CCR cut-off values varied across cohorts, reflecting population heterogeneity and differences in analytical methodologies. Therefore, no standardized clinical threshold can currently be recommended. Second, all included studies were conducted in East Asian populations, which may limit generalizability to non-Asian populations. Third, all studies were retrospective, and most were single-center in design. Fourth, reconstructed IPD was derived from published KM curves and did not include patient-level renal function, tumor stage, performance status, inflammatory markers, nutritional status, treatment covariates, or sex-stratified survival estimates. Therefore, multivariable Cox adjustment and sex-stratified reconstructed-IPD analyses were not feasible. Future research should establish standardized, sex-specific CCR cut-offs, examine renal-function-adjusted and multivariable models, and validate the prognostic utility of CCR in prospective multicenter cohorts.

## 5 Conclusion

Pretreatment CCR showed an association with overall survival in digestive tract cancers. However, standardized clinical thresholds, independence from renal function and other confounders, and generalizability to broader populations require validation in further prospective studies.

## Data Availability

The original contributions presented in this study are included in the article/Supplementary material, further inquiries can be directed to the corresponding authors.
