# Supplementary material for: Pretreatment creatinine-to-cystatin c ratio as a prognostic marker in digestive tract cancers: a meta-analysis with reconstructed individual patient data and trial sequential analysis
Source: Front Nutr. 2026 Jul 24;13:1834663. doi: 10.3389/fnut.2026.1834663 (PMC13447211; doi:10.3389/fnut.2026.1834663)
Supplement: Supplementary file 1 [file Table_1.docx]

**Supplementary Tables and Figures**

**Supplementary Table S1. Database Search Strategies**

| **Database** | **Records in original flow** | **Search date/window** | **Strategy** |
| --- | --- | --- | --- |
| PubMed | 80 | From database inception through 22 Oct 2025 (searched 22 Oct 2025) | ("creatinine/cystatin C ratio" OR "creatinine to cystatin C ratio" OR "creatinine over cystatin C ratio" OR "creatinine-to-cystatin C ratio" OR "sarcopenia index" OR "creatinine-cystatin c ratio") AND (carcinoma OR cancer OR tumor OR tumour OR neoplasm) |
| Web of Science | 163 | From database inception through 22 Oct 2025 (searched 22 Oct 2025) | TS=("creatinine/cystatin C ratio" OR "creatinine to cystatin C ratio" OR "creatinine over cystatin C ratio" OR "creatinine-to-cystatin C ratio" OR "sarcopenia index" OR "creatinine-cystatin c ratio") AND TS=(carcinoma OR cancer OR tumor OR tumour OR neoplasm) |
| Embase | 94 | From database inception through 22 Oct 2025 (searched 22 Oct 2025) | ("creatinine/cystatin C ratio":ti,ab,kw OR "creatinine to cystatin C ratio":ti,ab,kw OR "creatinine over cystatin C ratio":ti,ab,kw OR "creatinine-to-cystatin C ratio":ti,ab,kw OR "sarcopenia index":ti,ab,kw OR "creatinine-cystatin c ratio":ti,ab,kw) AND (carcinoma:ti,ab,kw OR cancer:ti,ab,kw OR tumor:ti,ab,kw OR tumour:ti,ab,kw OR neoplasm:ti,ab,kw) |
| Cochrane Library | 3 | From database inception through 22 Oct 2025 (searched 22 Oct 2025) | ("creatinine/cystatin C ratio" OR "creatinine to cystatin C ratio" OR "creatinine over cystatin C ratio" OR "creatinine-to-cystatin C ratio" OR "sarcopenia index" OR "creatinine-cystatin c ratio") AND (carcinoma OR cancer OR tumor OR tumour OR neoplasm) |

**Supplementary Table S2. PRISMA Flow Numbers**

| **Stage** | **N** | **Details** |
| --- | --- | --- |
| Records identified from databases | 340 | PubMed 80; Web of Science 163; Embase 94; Cochrane Library 3 |
| Duplicates removed | 108 | Calculated from original flow chart: 340 records identified minus 232 after de-duplication |
| Records screened after de-duplication | 232 | Title/abstract screening |
| Full-text articles assessed for eligibility | 19 | Full-text review |
| Full-text articles excluded | 10 | CCR grouped by quartiles (n=2); HR/95% CI not reported (n=2); not digestive-system tumors (n=6) |
| Studies included in qualitative and quantitative synthesis | 9 | 9 independent cohorts, 3,073 patients |

**Supplementary Table S3. Detailed Covariate Extraction**

| **Ref** | **Study** | **PMID/DOI** | **Adjusted covariates / model (original cohort)** |
| --- | --- | --- | --- |
| 11 | Feng 2025 | PMID 41041331; doi:10.3389/fimmu.2025.1645874 | Vessel invasion, perineural invasion, tumor length, ypT stage, ypN stage, pCR |
| 22 | Gao 2023 | PMID 37409249; doi:10.3389/fonc.2023.1155520 | Age, BMI, T stage, N stage, M stage, Perineural invasion, vascular invasion, pathological type, differentiation, size, CEA. |
| 23 | Sun 2022 | PMID 35045814; doi:10.1186/s12876-022-02093-4 | Age, BMI, pTNM stage, and tumor size. |
| 24 | Ji 2024 | PMID 38665913; doi:10.3389/fimmu.2024.1364728 | ECOG PS, No. of previous therapies, ascites, SATI, VATI, SMI, CT-determined sarcopenia. |
| 25 | Liu 2023 | PMID 37864250; doi:10.1186/s12967-023-04628-z | Hazard ratios were extracted directly from Kaplan-Meier survival curves and represent unadjusted estimates. |
| 26 | Zheng 2022 | PMID 35287579; doi:10.1186/s12877-022-02925-8 | The Cox regression was reported to be multivariable; however, the comprehensive list of adjustment covariates was not provided. |
| 27 | Fukushima 2024 | PMID 38683456; doi:10.1007/s10147-024-02539-7 | Age, CEA, ALB, lymph node metastasis, operative time, blood loss. |
| 28 | Liu 2024 | PMID 38706605; doi:10.3389/fonc.2024.1233768 | Lymph-node metastasis, ALB, CA125, WBC, neutrophil, monocyte, HB, TBIL, DBIL, alkaline phosphatase, uric acid, myosteatosis. |
| 29 | Bi 2024 | PMID 38319516; doi:10.1245/s10434-024-14969-8 | Perineural invasion, vascular invasion, tumor stage, adjunct treatment, CA199, myosteatosis |

**Supplementary Table S4. NOS Domain-Level Scores**

| **Ref** | **Study** | **Selection** | **Comparability** | **Outcome** | **Total** |
| --- | --- | --- | --- | --- | --- |
| 11 | Feng 2025 | 4 | 2 | 2 | 8 |
| 22 | Gao 2023 | 4 | 2 | 3 | 9 |
| 23 | Sun 2022 | 4 | 2 | 2 | 8 |
| 24 | Ji 2024 | 3 | 2 | 2 | 7 |
| 25 | Liu 2023 | 3 | 1 | 2 | 6 |
| 26 | Zheng 2022 | 4 | 2 | 2 | 8 |
| 27 | Fukushima 2024 | 4 | 2 | 2 | 8 |
| 28 | Liu 2024 | 3 | 2 | 2 | 7 |
| 29 | Bi 2024 | 4 | 2 | 2 | 8 |

**Supplementary Table S5. QUIPS Domain-Level Risk-of-Bias Assessment**

Risk of bias in included prognostic cohorts was assessed using the Quality In Prognostic Studies (QUIPS) tool (Hayden JA et al., Ann Intern Med 2013). Two authors independently reviewed all nine full-text articles and assigned domain-level judgments of Low, Moderate, or High risk of bias. Discrepancies were resolved by discussion.

| **Ref** | **Study** | **Participation** | **Attrition** | **Prognostic factor measurement** | **Outcome measurement** | **Study confounding** | **Statistical analysis & reporting** | **Overall** |
| --- | --- | --- | --- | --- | --- | --- | --- | --- |
| 11 | Feng 2025 | Moderate | Moderate | Moderate | Low | Moderate | Moderate | Moderate |
| 22 | Gao 2023 | Moderate | Moderate | Moderate | Low | Moderate | Moderate | Moderate |
| 23 | Sun 2022 | Moderate | Moderate | Moderate | Low | Moderate | Moderate | Moderate |
| 24 | Ji 2024 | Moderate | Moderate | Moderate | Moderate | Moderate | Moderate | Moderate |
| 25 | Liu 2023 | Moderate | Moderate | Moderate | Moderate | **High** | **High** | **High** |
| 26 | Zheng 2022 | Moderate | Moderate | Moderate | Moderate | Moderate | Moderate | Moderate |
| 27 | Fukushima 2024 | Moderate | Moderate | Moderate | Low | Moderate | Moderate | Moderate |
| 28 | Liu 2024 | Moderate | Moderate | Moderate | Low | Moderate | Moderate | Moderate |
| 29 | Bi 2024 | Moderate | Moderate | Moderate | Low | Moderate | Moderate | Moderate |

**Supplementary Table S6. Summary of exploratory meta-regression analyses**

| **Supplementary Table S6. Summary of exploratory meta-regression analyses** | | | | | |
| --- | --- | --- | --- | --- | --- |
| Moderators | Items | HR | 95% CI | P | Overall model P |
| Publication year | 2022 | Reference | Reference | Reference | 0.977 |
|  | 2023 | 0.873 | 0.301-2.534 | 0.756 |  |
|  | 2024 | 0.983 | 0.356-2.717 | 0.968 |  |
|  | 2025 | 1.021 | 0.269-3.876 | 0.97 |  |
| Sample size | N< 215 | Reference | Reference | Reference | - |
|  | >=215 | 0.851 | 0.485-1.491 | 0.517 |  |
| CCR cut-off type | higher | Reference | Reference | Reference | 0.157 |
|  | lower | 1.444 | 0.827-2.521 | 0.158 |  |
|  | unknown | 1.588 | 0.863-2.922 | 0.113 |  |
| Cancer type | biliary tract cancer | Reference | Reference | Reference | 0.606 |
|  | esophageal | 0.852 | 0.247-2.934 | 0.752 |  |
|  | gastrointestinal | 0.701 | 0.242-2.036 | 0.431 |  |
|  | pancreatic | 0.556 | 0.168-1.846 | 0.264 |  |

**Supplementary Figure S1. Tumour-Type Subgroup Forest Plot**

**Supplementary Figure S1. Forest plot of tumour-type subgroup analyses for the association between CCR level and overall survival (OS).**

**Supplementary Figure S2. QUIPS Risk-of-Bias Summary**
